# Multivariate analysis of genomics data to identify potential pleiotropic genes for type 2 diabetes, obesity and dyslipidemia using Meta-CCA and gene-based approach

**DOI:** 10.1371/journal.pone.0201173

**Published:** 2018-08-15

**Authors:** Yuan-Cheng Chen, Chao Xu, Ji-Gang Zhang, Chun-Ping Zeng, Xia-Fang Wang, Rou Zhou, Xu Lin, Zeng-Xin Ao, Jun-Min Lu, Jie Shen, Hong-Wen Deng

**Affiliations:** 1 Department of Endocrinology and Metabolism, The Third Affiliated Hospital of Southern Medical University, Guangzhou, GuangDong, PR China; 2 Center for Bioinformatics and Genomics, Department of Global Biostatistics and Data Science, School of Public Health and Tropical Medicine, Tulane University, New Orleans, Louisiana, United States of America; 3 School of Basic Medical Sciences, Central South University, Changsha, HuNan, PR China; University of Texas Health Science Center at San Antonio, UNITED STATES

## Abstract

Previous studies have demonstrated the genetic correlations between type 2 diabetes, obesity and dyslipidemia, and indicated that many genes have pleiotropic effects on them. However, these pleiotropic genes have not been well-defined. It is essential to identify pleiotropic genes using systematic approaches because systematically analyzing correlated traits is an effective way to enhance their statistical power. To identify potential pleiotropic genes for these three disorders, we performed a systematic analysis by incorporating GWAS (genome-wide associated study) datasets of six correlated traits related to type 2 diabetes, obesity and dyslipidemia using Meta-CCA (meta-analysis using canonical correlation analysis). Meta-CCA is an emerging method to systematically identify potential pleiotropic genes using GWAS summary statistics of multiple correlated traits. 2,720 genes were identified as significant genes after multiple testing (Bonferroni corrected *p* value < 0.05). Further, to refine the identified genes, we tested their relationship to the six correlated traits using VEGAS-2 (versatile gene-based association study-2). Only the genes significantly associated (Bonferroni corrected *p* value < 0.05) with more than one trait were kept. Finally, 25 genes (including two confirmed pleiotropic genes and eleven novel pleiotropic genes) were identified as potential pleiotropic genes. They were enriched in 5 pathways including the statin pathway and the PPAR (peroxisome proliferator-activated receptor) Alpha pathway. In summary, our study identified potential pleiotropic genes and pathways of type 2 diabetes, obesity and dyslipidemia, which may shed light on the common biological etiology and pathogenesis of these three disorders and provide promising insights for new therapies.

## Introduction

Type 2 diabetes is a serious chronic metabolic disorder characterized by hyperglycemia, insulin resistance (IR), destruction of pancreatic beta cells and impairment in insulin secretion [1]. Obesity, another serious universal health problem characterized by excess visceral fat and high Waist-Hip Ratio (WHR)) and general obesity (defined as having a Body Mass Index (BMI) of 25 or higher, is related to several chronic diseases including type 2 diabetes [2].(Both type 2 diabetes and obesity are associated with dyslipidemia [2, 3], which is characterized by hypertriglyceridemia, hypercholesterolemia, decreased HDL (high-density lipoprotein) and/or increased LDL (low-density lipoprotein). The coexistence of the three diseases is common among the populations, which increases the prevalence of other serious fatal diseases such as CVD (cardiovascular diseases) [4, 5] and stroke [6–8].

These three disorders are closely connected. Firstly, clinical observation and epidemiological data show that a number of type 2 diabetes patients are obese with dyslipidemia before receiving intervention or therapy [9]. Secondly, these three disorders have common risk factors and pathophysiological bases. For instance, a long term high fat diet is a significant risk factor for type 2 diabetes [10], obesity and dyslipidemia [2]. IR is one of the common pathophysiological bases for these three diseases [1, 11]. Moreover, previous studies have reported genetic correlations among these three disorders and indicated that many genes have pleiotropic effects [12–14]. For example, the SREBF1 gene was identified as a pleiotropic gene in the progression of type 2 diabetes, obesity, and dyslipidemia [12]. In addition, common pathways that could influence these three disorders were discovered in recent years, such as the AMPK signal pathway (AMP-activated protein kinase signal pathway) and the JNK-1 pathway [11, 15, 16].

The pleiotropic genes and pathways of the three diseases can partially explain the common biological pathogenesis of the three diseases. It is essential to identify pleiotropic genes that exert their influence on potentially common biological etiology and pathogenesis of these three disorders using systematic analysis approaches. Pleiotropic genes and their effects have been successfully identified in bivariate analyses of type 2 diabetes with obesity, type 2 diabetes with dyslipidemia, and obesity with dyslipidemia. Hasstedt *et al* [17] performed bivariate analyses of type 2 diabetes with BMI and type 2 diabetes with WHR, which identified significant pleiotropy loci (chromosome 13 at 26–30 MB) of type 2 diabetes with both. Li *et al* [3] found a stronger correlation between dyslipidemia associated genes (*APOB*, *APOE-C1-C2*, *CETP*, *CYP7A1 GCKR*, *MLXIPL*, *PLTP*, *TIMD4*) and glycemic traits including FG (fasting glucose) and HOMA-IR (homeostasis model assessment for IR, an important index for evaluating IR, calculated by FG* FI(fasting insulin)/22.5), which revealed the pleiotropic effects of dyslipidemia-associated genes on glycemic traits. Despite the bivariate analyses of these three diseases which have identified pleiotropic genes in recent years, multivariate analyses have not yet been performed. Undertaking multivariate analysis is desirable because it allows us to more systemically explore the common underlying genetic architecture and common etiology of these three disorders. Another important advantage of multivariate analysis is that it increases the statistical power for identifying associated genes exerting influences on multiple traits, which leads to more novel insights for drug gable gene targets compared to univariate and bivariate analyses [18].

Meta-CCA (meta-analysis using canonical correlation analysis) [19], a new systematical multivariate analysis tool recently proposed by Anna Cichonska, allows multivariate analyses between multiple SNPs and multiple traits [19], which enriches the pleiotropic information by combining correlation signals among multiple traits.

Inspired by this, we performed the current work to identify potential pleiotropic genes for type 2 diabetes, obesity, and dyslipidemia using Meta-CCA. We used six correlated quantitative traits reported to be established related factors for type 2 diabetes, obesity or dyslipidemia, including FG, FI, BMI, WHR, HDL and triglyceride (TG). Interestingly, our findings indicated that five pathways and 25 genes (including two confirmed pleiotropic genes and eleven novel pleiotropic genes) were potential pleiotropic genes for type 2 diabetes, obesity, and dyslipidemia. These findings yielded some genetic basis for the common biological etiology and pathogenesis, and thus provided promising insights for a potential common therapy for the three disorders.

## Materials and methods

### GWAS datasets and processing

#### Step1: Annotation genes to SNPs, and SNP prune

The large-scale GWAS datasets of the six correlated traits in the present study were downloaded from http://diagram-consortium.org/2015_ENGAGE_1KG/ [20, 21]. The glycemic traits (FG and FI) data [20] were derived from a meta-analysis of 13 original GWAS studies, including FG data for 46,694 individuals and FI data for 24,245 individuals. The obesity-related data (BMI and WHR) [20] were derived from a meta-analysis of 22 original GWAS studies, including BMI data for 87,048 individuals and WHR data for 54,572 individuals. The lipids (HDL and TG) data [21] were derived from a meta-analysis of 22 original GWAS studies, including 62,166 individuals for both HDL and TG. The large-scale GWAS datasets were collected by the European Network for Genetic and Genomic Epidemiology (ENGAGE) Consortium, and all samples were from individuals of European ancestry **(The details were shown in** Table 1**)**. The large-scale GWAS datasets were the largest datasets that included all six correlated traits and contained all the information needed to conduct the analyses in the Meta-CCA framework in one ethnicity (Caucasians). We selected the overlapped SNPs (9,411,134 SNPs) of the six traits to perform the multivariate analysis.

**Table 1:** Details and phenotypic pearson correlation coefficients of the six traits in European ancestry.

Traits	Number of SNPs	Number of individuals	The phenotypic correlation structures between traits					
			FG	FI	BMI	WHR	HDL	TG
FG	9,967,161	46,694	1	0.35	0.24	0.17	-0.15	0.19
FI	9,837,043	24,245	0.35	1	0.52	0.39	-0.37	0.40
BMI	9,953,164	87,048	0.24	0.52	1	0.51	-0.32	0.30
WHR	9,954,793	54,572	0.17	0.39	0.51	1	-0.30	0.33
HDL	9,549,054	62,166	-0.15	-0.37	-0.32	-0.30	1	-0.52
TG	9,544,498	62,166	0.19	0.40	0.30	0.33	-0.52	1

FG Stands for: Fasting glucose.

FI Stands for: Fasting insulin.

BMI Stands for: Body Mass Index.

WHR Stands for: Waist-Hip Ratio.

HDL Stands for: High-density lipoprotein.

TG Stands for: Triglyceride.

**Fig 1:**
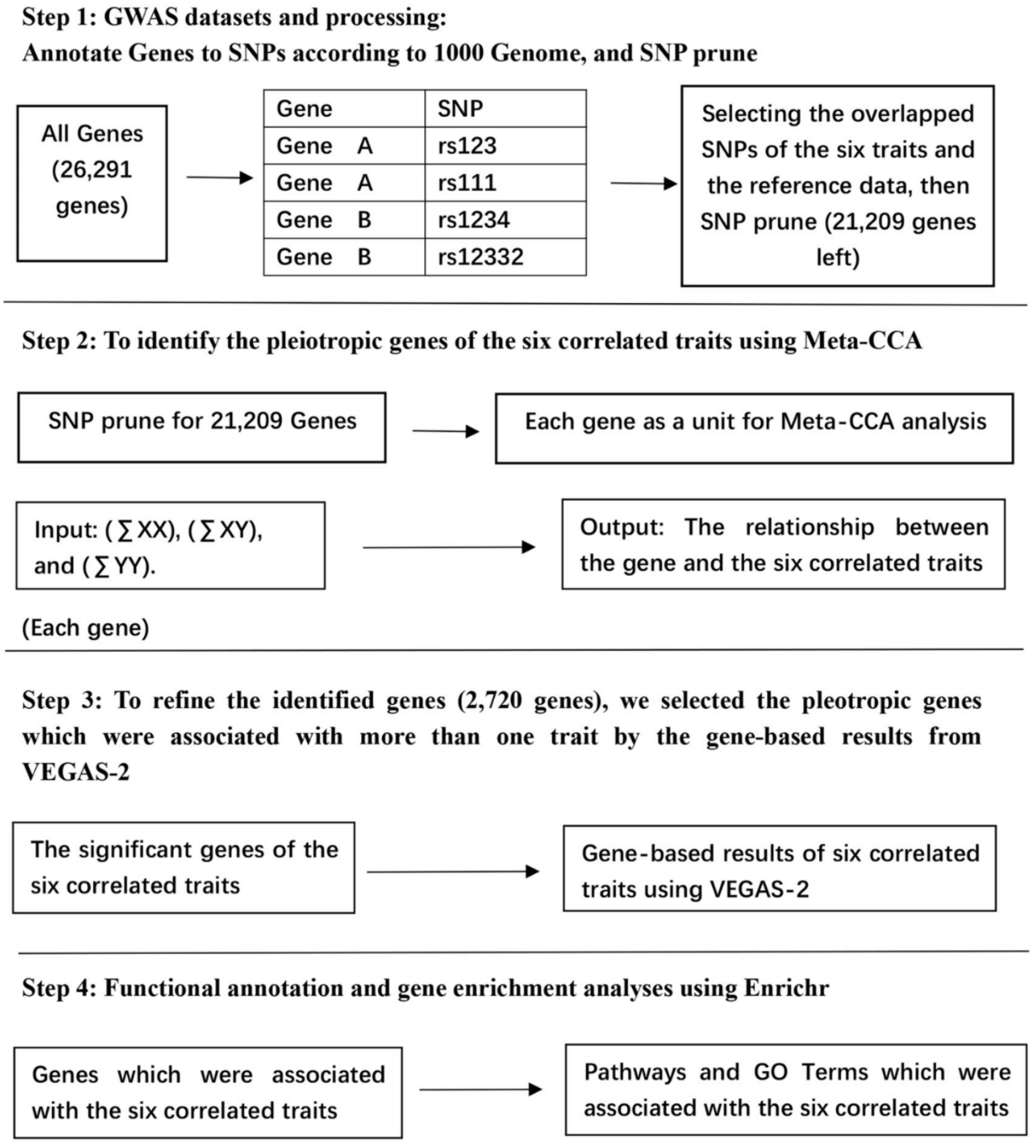
The analytical workflow of the present work.

The analytical workflow of our study is presented in Fig 1. Firstly, we completed the gene annotation according to the 1000 Genome datasets using PLINK1.9. We downloaded the reference data, which contained 26,291 genes from the website: https://www.cog-genomics.org/static/bin/plink/glist-hg19. We recognized the transcript including all SNPs (both exonic and intronic SNPs) in the region as genes. We selected the overlapped SNPs between the six traits and the reference data in our study. After the gene annotation of SNPs, we pruned the SNPs for each gene using the parameter r^2^ = 0.01 [22]. r is the Pearson correlation coefficient between any of the two SNPs in one region. The purpose of SNP pruning is to reduce potential biases caused by the linkage disequilibrium (LD) among SNPs [22].

For the present work, the GWAS datasets we used provided not only the *p* value of the SNPs but also the regression coefficient β and the standard error (SE). We normalized the regression coefficient β before conducting the Meta-CCA using the following equation (n is the sample size in the corresponding GWAS dataset of each trait):

$$\beta^{normal}=\frac{\beta }{\sqrt{n}*SE}$$ (1)

### Statistical analysis

#### Step 2: Meta-CCA analysis

The present work was conducted by the Meta-CCA R package, and each gene as a unit for Meta-CCA analysis. We recognized the transcript including all SNPs (both exonic and intronic SNPs) in the region as genes. The program of Meta-CCA required three basic data inputs: the genotypic correlation structures between SNPs (∑XX), the correlation coefficients between SNPs and traits (∑XY), and the phenotypic correlation structures between traits (∑YY) (Table 1) [19]. In Meta-CCA, ∑XX was estimated using a reference SNP dataset such as the HapMap data or the 1000 Genomes data representing the study population. It is better if ∑XX was estimated from the target population or the same ethnicity from the database used [19]. As all of the participants in our study were Caucasians, we calculated the ∑XX based on the 1000 Genomes data and downloaded the reference data from 1000 Genomes Project (Phase 3 European (CEU) population reference data (https://www.cog-genomics.org/static/bin/plink/glist-hg19). ∑XY was estimated by the normalized regression coefficient β:

$$\sum XY=\frac{X^{T}Y}{N-1}=\left(\begin{array}{cccc} \beta_{11} & \beta_{12} & \cdots & \beta_{1P}\\ \beta_{21} & \beta_{22} & \cdots & \beta_{2P}\\ \vdots & \vdots & \ddots & \vdots \\ \beta_{G1} & \beta_{G2} & \cdots & \beta_{GP} \end{array}\right)$$ (2)

G and P are the number of genotypic and phenotypic variables, respectively.

And ∑YY was estimated based on the Phenotypic Pearson Correlation Coefficients (YY), which was shown in Table 1 [23]. In the original study, all of the participants had the clinical data of the six traits (FG, FI, BMI, WHR, HDL and TG). The phenotypic Pearson correlation coefficients between the six traits were calculated from the same corresponding individuals [23]. The details of the exact procedures of Meta-CCA program were described in Anna Cichonska’s paper [19]. After Meta-CCA, we obtained the output data of the relationship between the genes and the six traits. We used a p value < 0.05 (after Bonferroni correction) as the nominal significance threshold.

#### Step 3: Gene-based association analysis

To refine the identified genes by Meta-CCA, we tested their specific relationships with the six traits respectively using VEGAS-2 (Versatile Gene-based Association Study–2) [24, 25], a gene-based algorithm widely used for gene-based *p* value calculation using GWAS summary statistics. VEGAS-2, an approach provides the correlation of all the SNPs in one gene region for one single trait, also has lower false positive rates compared with other gene-based approaches [26, 27]. We selected the potential pleiotropic genes significantly associated with more than one trait (P value< 0.05/2720, Bonferroni correction) [28] after obtaining the gene-based p-value of each gene for the six traits using VEGAS-2.

### Functional annotation and gene enrichment analyses

#### Step 4: Pathway and GO (Gene Ontology) term enrichment analyses of the potential pleiotropic genes

To explore the biological functions of the identified potential pleiotropic genes, we performed pathway enrichment analyses and GO enrichment analyses for the potential pleiotropic genes using Enrichr (a web server tool for gene set enrichment analysis: http://amp.pharm.mssm.edu/Enrichr/) [29]. The Pathway and GO Term enrichment analyses also provide a better understanding of the polygenic associations and the potential mechanisms of the biological process. With this program, we used hypergeometric tests and Fisher’s exact tests for the statistical analysis. Benjamini-Hochberg corrected *p* value <0.05 in the enrichment analysis is used as the threshold for significance.

## Results

After gene annotation and SNP pruning, 21,209 genes were left to conduct the Meta-CCA analysis in our study. The number of SNPs in each gene ranged from 1 to 280; the average was 13. We used the threshold of 0.05/21209 (Bonferroni correction) as our target alpha level for the Meta-CCA analysis [28]. For the Meta-CCA analysis, 2,720 genes with the p value < 0.05/21209 were identified as potential pleiotropic genes for the six correlated quantitative traits. After Meta-CCA analysis, we tested the 2,720 genes’ relationship to the six traits using VEGAS-2. Only the genes significantly associated (Bonferroni corrected p value < 0.05) with more than one trait were kept [24, 25]. There were 31, 0, 75, 1, 225 and 185 significant genes (Bonferroni corrected *p* value < 0.05) for FG, FI, BMI, WHR, HDL, and TG respectively. By screening the genes based on the results of gene-based *p* value, a total of 25 associated genes related to more than one trait in VEGAS-2 analysis were identified as potential pleiotropic genes for type 2 diabetes, obesity and dyslipidemia. The details are shown in Table 2.

**Table 2:** The features of the significant potential pleiotropic genes.

Genes	r*	P-value for one single trait					
		BMI^#^	WHR^#^	FG^#^	FI^#^	HDL^#^	TG^#^
*SIK3*^a^[30, 31]	2.63E-01	6.08E-01	9.69E-02	4.19E-01	5.79E-01	1.00E-06	1.00E-06
*CETP*^a^[3, 32]	2.48E-01	2.70E-01	4.04E-01	5.90E-01	6.25E-01	1.00E-06	1.00E-06
*LIPC*^a^[33, 34]	2.27E-01	2.02E-01	6.78E-01	3.58E-02	2.05E-01	1.00E-06	1.00E-06
*GALNT2*^a^[21, 35]	2.05E-01	1.93E-01	6.87E-01	6.66E-01	3.09E-01	1.00E-06	1.00E-06
*SNX17*^a^[36]	1.10E-01	3.08E-01	6.89E-03	6.00E-06	1.19E-03	9.79E-02	1.00E-06
*GCKR*^b^[21, 37, 38]	9.73E-02	2.06E-01	6.89E-03	1.00E-06	6.10E-05	1.09E-01	1.00E-06
*LIPC-AS1*^c^	9.07E-02	5.01E-01	7.92E-01	1.09E-01	1.71E-01	1.00E-06	1.00E-06
*LPL*^a^[39, 40]	9.05E-02	5.51E-01	3.83E-01	1.57E-01	6.69E-01	1.00E-06	1.00E-06
*HLA-DQA1*^d^[21, 41]	8.42E-02	6.47E-01	2.18E-01	4.26E-01	8.84E-01	1.20E-05	1.00E-06
*IFT172*^c^	8.40E-02	3.57E-01	6.32E-03	4.00E-06	3.13E-04	8.69E-02	1.00E-06
*KRTCAP3*^c^	8.28E-02	3.30E-01	7.51E-03	1.00E-05	1.46E-03	1.30E-01	1.00E-06
*CSGALNACT1*^c^	8.26E-02	6.89E-02	7.66E-01	6.93E-01	2.44E-01	1.00E-05	1.00E-06
*APOA5*^a^[42, 43]	8.23E-02	5.21E-01	3.53E-01	2.22E-01	4.35E-01	1.00E-06	1.00E-06
*EIF2B4*^c^	7.28E-02	3.48E-01	7.18E-03	5.00E-06	1.48E-03	7.09E-02	1.00E-06
*GTF3C2*^c^	7.07E-02	4.26E-01	1.19E-02	1.20E-05	1.61E-03	7.19E-02	1.00E-06
*ZNF513*^c^	7.00E-02	3.19E-01	6.02E-03	1.10E-05	1.41E-03	9.69E-02	1.00E-06
*NRBP1*^c^	6.21E-02	3.22E-01	9.40E-03	9.00E-06	1.60E-03	1.46E-01	1.00E-06
*FNDC4*^c^	6.19E-02	3.39E-01	5.16E-03	1.00E-06	1.00E-04	1.02E-01	1.00E-06
*APOA1*^a^[44, 45]	5.52E-02	5.43E-01	2.43E-01	3.54E-01	4.51E-01	1.00E-06	1.00E-06
*FADS1*^b^[21, 46, 47]	5.22E-02	8.79E-02	5.49E-01	3.00E-06	6.44E-01	1.00E-06	1.00E-06
*TMEM258*^c^	5.15E-02	7.69E-02	6.77E-01	1.00E-06	7.05E-01	1.00E-06	1.00E-06
*FEN1*^d^[21, 48]	4.82E-02	1.35E-01	6.67E-01	1.00E-06	6.54E-01	1.00E-06	1.00E-06
*ZPR1*^d^[21, 49]	4.36E-02	5.01E-01	3.51E-01	2.39E-01	4.41E-01	1.00E-06	1.00E-06
*APOC2*^d^[21, 50]	4.04E-02	1.10E-01	5.32E-01	6.99E-02	7.43E-01	1.00E-06	1.00E-06
*CLPTM1*^c^	3.98E-02	1.96E-01	6.07E-01	9.79E-02	7.72E-01	1.00E-06	1.00E-06

* Stands for: Canonical correlation value for the six correlated traits which is the result of Meta-CCA.

^#^ Stands for: P-value for each trait which is the result of gene-based analysis.

^a^ Stands for: This gene hasn’t been identified by any previous GWAS studies for type 2 diabetes and obesity, but has been reported to be associated with hyperglycemia, obesity and dyslipidemia in other types of previous studies.

^b^ Stands for: This gene was previously reported to be associated with type 2 diabetes, obesity and dyslipidemia, which was confirmed by our present study.

^c^ Stands for: Novel pleotropic gene for type 2 diabetes, obesity and dyslipidemia.

^d^ Stands for: This gene hasn’t been identified by any previous studies for obesity, but has been reported to be associated with type 2 diabetes and dyslipidemia in previous study.

Interestingly, four of the top five significant genes (*GALNT2*, *SNX17*, *CETP*, *LIPC*) were regarded as dyslipidemia associated genes in the original GWAS study [21]. In particular, two (*GALNT2*, *SNX17*) were also suggested to be associated with type 2 diabetes and obesity in previous studies [35, 36]. All 25 potential pleiotropic genes were identified as the associated genes/loci (with at least one SNP p value < 5*10^−8^) for TG in the original GWAS study, while eight of these 25 genes (*GALNT2*, *GCKR*, *LPL*, *FADS1*, *LIPC*, *CETP*, *APOA5*, *ZPR1*) were reported to be TG associated genes in the original GWAS study after validation [21]. Specifically, two of these 16 genes (*FADS1*, *GCKR*) have been identified as susceptibility candidate genes for type 2 diabetes in early GWAS studies [37, 47, 51].

For the results of the pathway enrichment analyses, significant enrichment was observed in five human pathways conforming to the up-to-date 2016 Wiki-pathway database (Table 3) [29, 52], such as Statin Pathway (WP430), Composition of Lipid Particles (WP3601), Triacylglyceride Synthesis (WP325), PPAR (Peroxisome proliferator-activated receptor) Alpha Pathway (WP2878), Fatty Acid Beta Oxidation (WP143). The most significant pathway was the statin pathway (WP430), which contains six potential pleiotropic genes (*CETP*, *LIPC*, *APOC2*, *APOA1*, *LPL*, *APOA5*), suggesting a close relationship between the statin pathway and the three disorders.

**Table 3:** Pathway enrichment analysis of the potential pleiotropic genes.

Term (Pathway)	P-value	Benjamini-Hochberg P-value	Genes
Statin Pathway(WP430)	1.44E-12	4.03E-10	*CETP*, *LIPC*, *APOC2*, *APOA1*, *LPL*, *APOA5*
Composition of Lipid Particles(WP3601)	1.44E-07	1.35E-06	*CETP*, *LPL*, *APOA1*
Triacylglyceride Synthesis(WP325)	4.07E-04	1.89E-03	*LIPC*, *LPL*
PPAR Alpha Pathway(WP2878)	4.79E-04	1.91E-03	*APOA1*, *APOA5*
Fatty Acid Beta Oxidation(WP143)	8.21E-04	2.55E-03	*LIPC*, *LPL*

GO enrichment analyses (conforming to the up-to-date 2017 database) [29, 52] revealed that the biological functions of these pleiotropic genes were mainly involved in the metabolism of lipids. For the GO biological process, the top five significant GO terms were Triglyceride homeostasis (GO:0070328), Cellular triglyceride homeostasis (GO:0035356), Positive regulation of lipoprotein lipase activity (GO:0051006), Cholesterol homeostasis (GO:0042632) and Reverse cholesterol transport (GO:0043691). For the GO cellular component, the top five significant GO terms were Very-low-density lipoprotein particle (GO:0034361), Spherical high-density lipoprotein particle (GO:0034366), Early endosome (GO:0005769), Early endosome lumen (GO:0031905) and Integral component of Golgi medial cisterna membrane (GO:1990703). For the GO molecular function, the top five significant GO terms were Intermembrane cholesterol transfer activity (GO:0120020), Cholesterol transporter activity (GO:0017127), Cholesterol binding (GO:0015485), Phosphatidylcholine-sterol O-acyltransferase activator activity (GO:0060228), and High-density lipoprotein particle receptor binding (GO:0070653). The results of the GO enrichment analysis are summarized in Table 4. GO Term is the gene collection of different arborescence types [53]. Therefore, some GO Term is the branch of others. As a result, there is a considerable overlap of genes between related GO-terms such as “Triglyceride homeostasis” and “Cellular triglyceride homeostasis”.

**Table 4:** Top five significant GO term enrichment analysis of the potential pleiotropic genes.

Term (GO Biological Process)	P-value	Benjamini-Hochberg P-value	Genes
Triglyceride homeostasis (GO:0070328)	3.19E-16	2.44E-13	*CETP*, *GCKR*, *LIPC*, *APOC2*, *LPL*, *APOA1*, *APOA5*
Cellular triglyceride homeostasis (GO:0035356)	2.21E-15	8.42E-13	*CETP*, *GCKR*, *LIPC*, *APOC2*, *LPL*, *APOA1*, *APOA5*
Positive regulation of lipoprotein lipase activity (GO:0051006)	2.29E-11	5.83E-09	*LIPC*, *APOC2*, *LPL*, *APOA1*, *APOA5*
Cholesterol homeostasis (GO:0042632)	3.06E-11	5.83E-09	*CETP*, *LIPC*, *APOC2*, *LPL*, *APOA1*, *APOA5*
Reverse cholesterol transport (GO:0043691)	1.58E-10	2.01E-08	*CETP*, *LIPC*, *APOC2*, *APOA1*, *APOA5*
Term (GO Cellular Component)	P-value	Benjamini-Hochberg P-value	Genes
Very-low-density lipoprotein particle (GO:0034361)	7.77E-07	1.17E-04	*APOC2*, *APOA1*, *APOA5*
Spherical high-density lipoprotein particle (GO:0034366)	4.18E-05	3.16E-03	*APOC2*, *APOA1*
Early endosome (GO:0005769)	8.43E-04	2.09E-02	*SNX17*, *APOC2*, *APOA1*
Early endosome lumen (GO:0031905)	1.11E-03	2.09E-02	*SNX17*, *APOC2*, *APOA1*
Integral component of Golgi medial cisterna membrane (GO:1990703)	1.08E-03	2.09E-02	*CSGALNACT1*, *GALNT2*
Term (GO Molecular Function)	P-value	Benjamini-Hochberg P-value	Genes
Intermembrane cholesterol transfer activity (GO:0120020)	9.56E-07	1.71E-04	*CETP*, *APOA1*, *APOA5*
Cholesterol transporter activity (GO:0017127)	4.95E-06	4.43E-04	*CETP*, *APOA1*, *APOA5*
Cholesterol binding (GO:0015485)	2.06E-05	6.69E-04	*CETP*, *APOA1*, *APOA5*
Phosphatidylcholine-sterol O-acyltransferase activator activity (GO:0060228)	2.24E-05	6.69E-04	*APOA1*, *APOA5*
High-density lipoprotein particle receptor binding (GO:0070653)	2.24E-05	6.69E-04	*APOA1*, *APOA5*

In summary, our present work identified twenty-five potential pleiotropic genes as well as the enriched pathways and GO terms of potential pleiotropic genes for type 2 diabetes, obesity and dyslipidemia.

## Discussion

**Fig 2:**
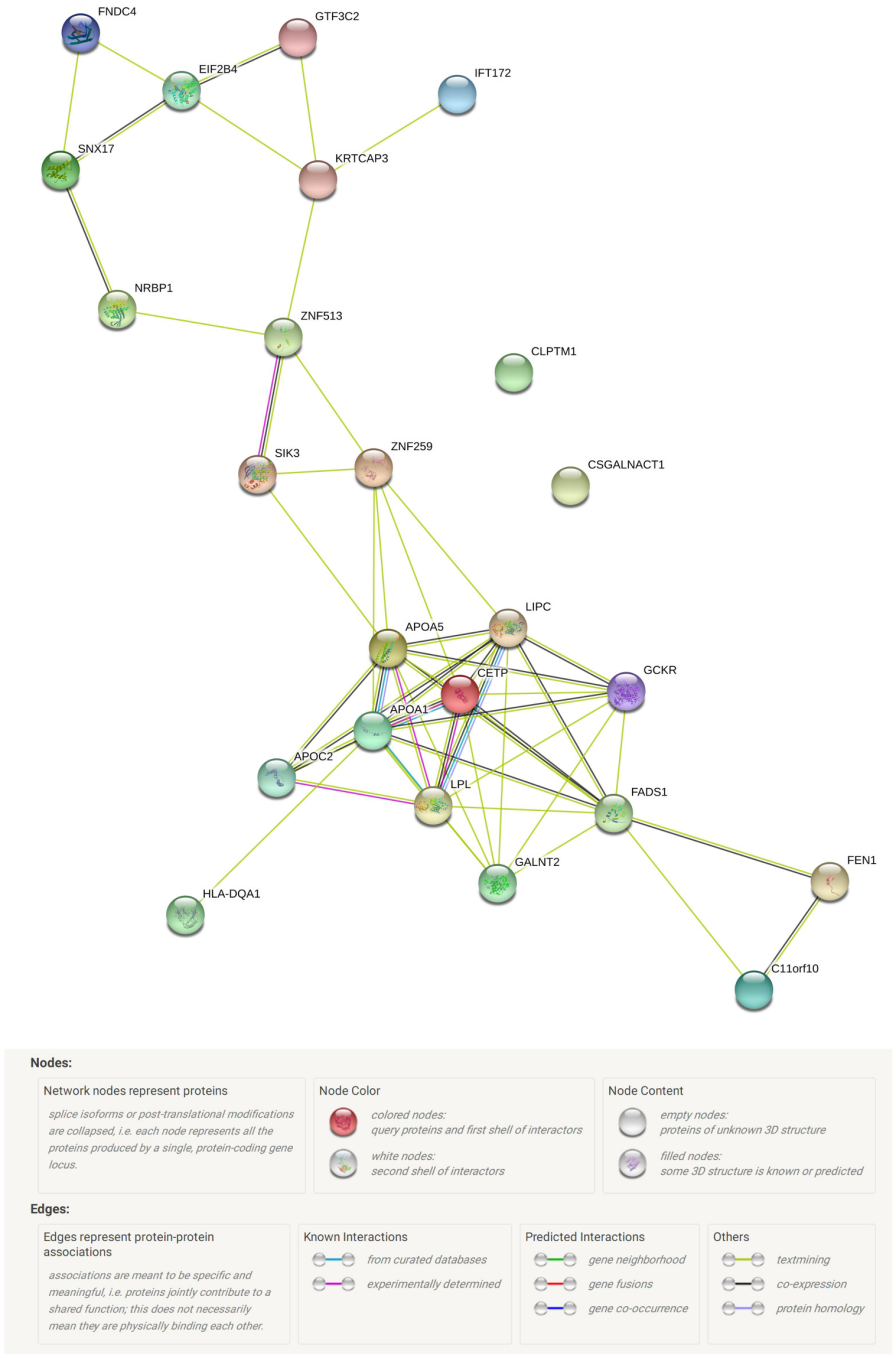
The nodes represent proteins which were encoded by corresponding genes, edges represent the protein-protein association, line color represents types of interaction evidence (e.g., text mining, co-expression and so on). All of the interacting proteins with an interaction score ≥ 0.15 (based on previous study).

The present study, the first systemically multivariate analysis of genomics data for type 2 diabetes, obesity and dyslipidemia jointly using Meta-CCA, identified potential pleiotropic genes as well as enriched pathways and GO terms. Importantly, two of the 25 identified genes (*GCKR*, *FADS1*) were reported to be associated with type 2 diabetes, obesity and dyslipidemia in different prior studies [21, 37, 38, 46, 47], validated by our present study. Other significant genes, excluding the genes that were reported to be associated with hyperglycemia, obesity and dyslipidemia in other types of previous studies, might be novel pleiotropic candidate genes (such as *LIPC-AS1*, *IFT172*, *KRTCAP3*, *CSGALNACT1*, *EIF2B4*, *GTF3C2*, *ZNF513*, *NRBP1*, *FNDC4*, *TMEM258*, and *CLPTM1*) for the three disorders. We preformed the functional protein-protein interaction network analysis for the potential pleiotropic candidate genes by using STRING 10.5 (https://string-db.org/cgi/input.pl). Fig 2 shows that there are interactions between most of the potential pleiotropic candidate genes. The results not only revealed some of the shared genetic components but also provided novel insights for exploring the potential common biological pathogenesis of these three disorders.

Many genes and pathways have pleiotropic effects on more than one disease, a common phenomenon supported by this study of type 2 diabetes, obesity and dyslipidemia. Recently, animal experiments and cross-sectional population-based studies have shown evidence of large shared gene components. Pleiotropic genes have been successfully identified in bivariate analyses of type 2 diabetes with obesity, type 2 diabetes with dyslipidemia, and obesity with dyslipidemia. However, multivariate analysis had not previously been conducted for these three disorders simultaneously. Systemically exploring the pleiotropic genes and their effects on these three disorders is essential, and is possible because of the accessibility of the GWAS summary statistics. The advantages of Meta-CCA are listed as follows. Firstly, Meta-CCA can detect correlations between multiple variants and multiple traits based on GWAS summary statistics [19], which might provide richer clues for finding novel gene targets in multivariate analyses compared to the univariate and bivariate analysis [18]. For example, TMEM258, a gene for adipose tissue regulation, was not identified by any previous GWAS studies for type 2 diabetes and obesity, but was one of the novel pleiotropic candidate genes for type 2 diabetes, obesity and dyslipidemia identified in this study. Secondly and notably, Meta-CCA can identify novel candidates, since some of the associations become detectable only when multiple variants and multiple traits are tested jointly [19]. For example, *CETP*, a well-known gene for dyslipidemia, was not identified by any previous GWAS studies for type 2 diabetes, but it was one of the novel findings in our study. Last but not least, Meta-CCA is a cost-effective analytical method based on the data of GWAS summary statistics, which provides an enlarged larger effective sample size to detect potential pleiotropic genes for multivariate traits. Meta-CCA and similar types of analyses are an emerging and powerful tool for detection of pleiotropic genes of multiple correlated traits using GWAS summary statistics.

Among the 25 potential pleiotropic genes, two genes, *GCKR*, and *FADS1*, were suggested to be pleiotropic genes for type 2 diabetes, obesity and dyslipidemia based on the results of previous studies [21, 37, 38, 46, 47]. *GCKR*, located in 2p23.3, which encodes the protein belonging to the glucosidase regulatory subfamily, which in turn inhibits glucosidase by binding to enzymes in pancreatic islet cells and liver. A recent study found that the variants of *GCKR* were associated with obesity in postmenopausal women [38]. *FADS1*, located in 11q12.2, encodes a protein that is a member of fatty acid desaturases. *FADS1* was reported to be related to type 2 diabetes by a previous GWAS study, but the mechanism was still unknown [51].

**Fig 3:**
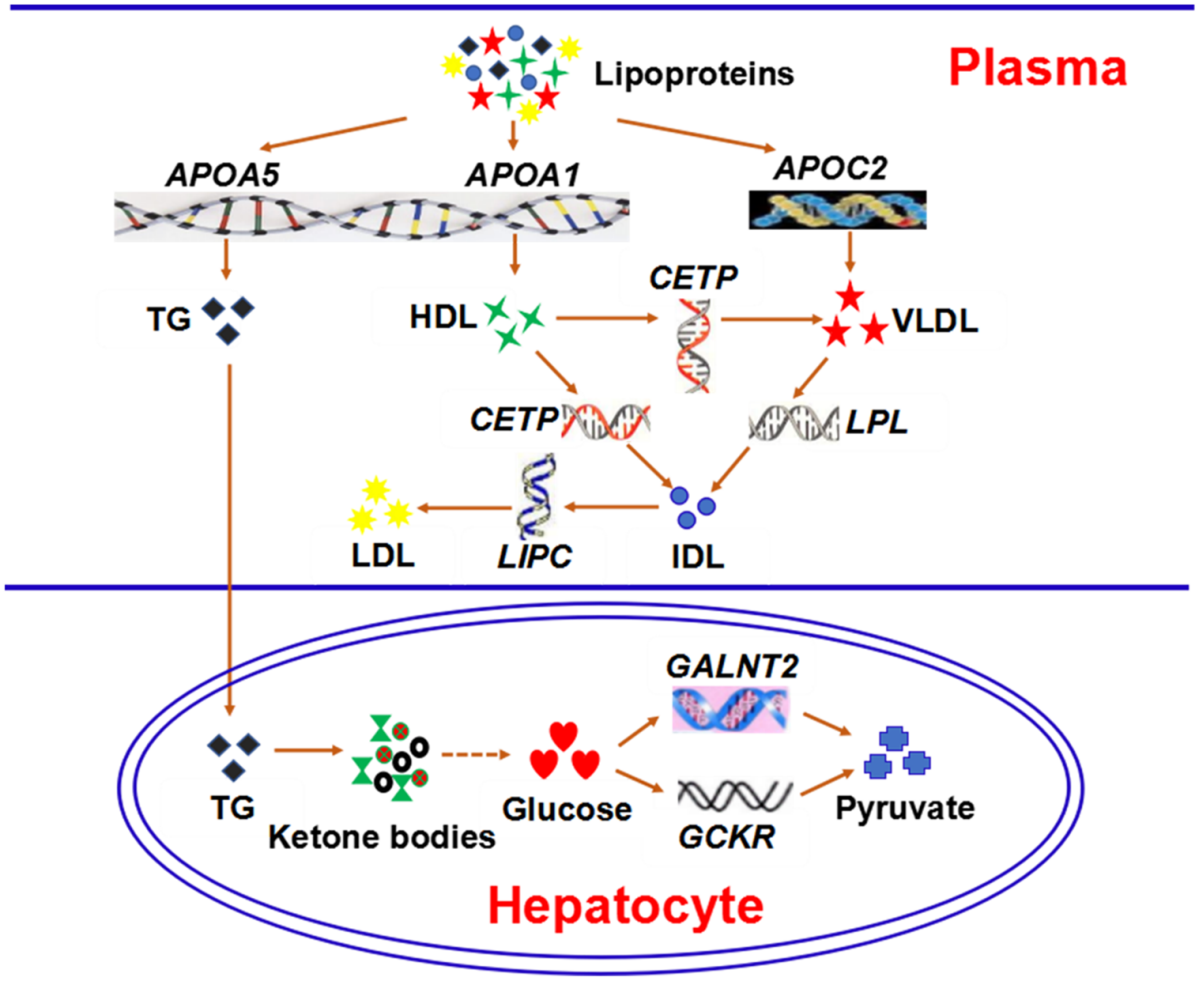
Eight potential pleiotropic genes (the italic) affected the three disorders through these important metabolic routes. From a biochemistry point of view, eight (*APOA5*, *APOA1*, *APOC2*, *CETP*, *LPL*, *LIPC*, *GCKR*, *GALNT2*) of the twenty-five potential pleiotropic genes were involved in important metabolic routes. *APOA5*, *APOA1*, and *APOC2* encode lipoproteins which mainly ferry TG, HDL, and VLDL, respectively. *CETP* encodes cholesteryl ester-transfer protein, which transfers HDL into VLDL and IDL by involving the transportation of cholesteryl ester. LPL is a lipoprotein lipase which plays a critical role in lipid metabolism such as transferring VLDL into IDL. The function of the protein hepatic triglyceride lipase encoded by *LIPC* is important in catabolism of lipids, including transferring IDL into LDL. *GALNT2* and *GCKR* are involved in the metabolism of glucose. The dotted line stands for the complex metabolic routes of gluconeogenesis.

From a biochemistry point of view, eight (*APOA5*, *APOA1*, *APOC2*, *CETP*, *LPL*, *LIPC*, *GCKR*, *GALNT2*) of the twenty-five potential pleiotropic genes were involved in important metabolic routes. Details are summarized in Fig 3. *APOA5*, *APOA1*, and *APOC2* encode lipoproteins which mainly ferry TG, HDL, and VLDL (very low density lipoprotein), respectively. *CETP* encodes cholesteryl ester-transfer protein, which transfers HDL into VLDL and IDL (intermediate density lipoprotein) by involving the transportation of cholesteryl ester. LPL is a lipoprotein lipase which plays a critical role in lipid metabolism such as transferring VLDL into IDL. The function of the protein hepatic triglyceride lipase encoded by *LIPC* is important in catabolism of lipids, including transferring IDL into LDL. *GALNT2* and *GCKR* are involved in the metabolism of glucose as mentioned above.

Our results, as previously described, have identified 25 genes and five pathways associated with type 2 diabetes, obesity, and dyslipidemia. Interestingly, all 25 genes were identified as the associated genes/loci for TG in the original GWAS study [21], though just eight of these 25 genes were refined in the validation stage [21]. All five pathways were associated with the metabolism of lipids. Specifically, two types of lipid-lowering drugs successfully targeted the statin pathway and PPAR Alpha pathway respectively, suggesting that abnormal plasma levels of lipids play a critical role in the common biological pathogenesis of the three disorders. Some drugs targeted on the statin pathway have been used successfully in therapy for type 2 diabetes patients with dyslipidemia. Another significant pathway is the PPAR Alpha pathway. The PPAR pathway family, which includes the PPAR Alpha pathway, the PPAR Beta pathway, and the PPAR Gamma pathway, plays a key role in substance metabolism (including glucose metabolism, lipid metabolism, and protein metabolism). Specifically, PPAR Alpha was a core factor for fatty acid oxidation in liver, which was activated by ligands or drugs such as fibrates, resulting in a decrease in serum level of TG [54]. PPAR Gamma was also an important factor for the etiology of IR [55]. Drugs targeting PPAR Gamma, such as thiazolidinedione, were effective in the control of IR [56].

Our present study also indicated that TG played an important role in these three disorders, as all 25 potential pleiotropic genes were identified as associated genes/loci for TG in the original GWAS study (though eight of these 25 genes were reported to be TG associated genes after validation) [21]. For type 2 diabetes, hypertriglyceridemia is the most common type of dyslipidemia [57], which is mainly induced by IR and impairment in insulin secretion. Further, genomic studies [57, 58] have indicated that hypertriglyceridemia has a higher genetic correlation with type 2 diabetes than other types of dyslipidemia. For obesity, most of the plasma TG is determined by the level of VLDL-TG (the balance between synthesis and clearance of VLDL-TG), and the synthesis of VLDL-TG is associated with total fat mass and liver fat [59]. Thus, the large amount of fat mass in obese patients leads to increasing synthesis of VLDL-TG, but the clearance of VLDL-TG remains unchanged. Hypertriglyceridemia is a principal characteristic of dyslipidemia and is linked to many other types of dyslipidemia such as decreased HDL level and increased small dense LDL level [60]. Above all, the metabolism of TG seems to play a core role in the common biological pathogenesis of these three disorders.

Our study not only provides a better understanding of the shared genetic background for the three disorders, but also produced a list of potential novel pleiotropic candidate genes for follow-up study in further biological experiments. Some of the 25 pleiotropic genes (*LIPC-AS1*, *IFT172*, *KRTCAP3*, *CSGALNACT1*, *EIF2B4*, *GTF3C2*, *ZNF513*, *NRBP1*, *FNDC4*, *TMEM258*, and *CLPTM1*) were first reported to be associated with type 2 diabetes, obesity and dyslipidemia. The findings of our present work were not completely consistent with the findings in previous GWAS studies or other types of systematically analysis studies in type 2 diabetes and other metabolic related diseases (The details of the overlapped identified genes and the novel potential pleiotropic candidate genes were shown in Table 2). The reason for the different findings in type 2 diabetes might be the using of different datasets and different methods in these studies. For example, one published work that using integrative omics data shown that 15 SNPs and the corresponding genes were associated with type 2 diabetes [61]. However, these genes were not identified by our present work. Our present work did not identify all the genes that were identified in GWASs or other types of studies, as it was only a supplementary study to identify the potential pleiotropic genes for chronic complex diseases. We hope that the potential novel pleiotropic candidate genes can provide some clues for molecular biologists performing future functional validation studies to determine whether the findings truly have pathophysiological significance for type 2 diabetes, obesity and dyslipidemia.

## Conclusion

In this study, we identified and assessed some potential pleiotropic genes and pathways for type 2 diabetes, obesity and dyslipidemia using novel Meta-CCA analysis. The findings validated two previously identified pleiotropic genes (*GCKR*, *FADS1*) for these three disorders and highlighted another eleven significant genes (*LIPC-AS1*, *IFT172*, *KRTCAP3*, *CSGALNACT1*, *EIF2B4*, *GTF3C2*, *ZNF513*, *NRBP1*, *FNDC4*, *TMEM258*, and *CLPTM1*) as potential novel pleiotropic candidate genes for the three disorders. Further, the potential pleiotropic genes were significantly enriched in five pathways including the statin pathway and PPAR Alpha pathway. In conclusion, our findings may yield novel insights into exploring the common biological pathogenesis of these three disorders, which ultimately may lead to the development of effective drug therapies.
